# “Hospitals respond to demand. Public health needs to respond to risk”: health system lessons from a case study of northern Queensland’s COVID-19 surveillance and response

**DOI:** 10.1186/s12913-023-10502-x

**Published:** 2024-01-18

**Authors:** Alexandra Edelman, Tammy Allen, Susan Devine, Paul F. Horwood, Emma S. McBryde, Julie Mudd, Jeffrey Warner, Stephanie M Topp

**Affiliations:** 1 Menzies School of Health Research, Charles Darwin University, Alice Springs, Northern Territory, Australia; 2 College of Public Health, Medical and Veterinary Sciences, James Cook University, Townsville, Queensland Australia; 3 Australian Institute of Tropical Health and Medicine, James Cook University, Townsville, Queensland Australia; 4 College of Medicine and Dentistry, James Cook University, Townsville, Queensland Australia; 5 James Cook University, Building 41, Level 2, 1 James Cook Drive, Douglas, Queensland 4811 Australia

**Keywords:** Public health, Health systems, Pandemic preparedness, COVID-19, Governance, Queensland, Australia

## Abstract

**Background:**

The vast region of northern Queensland (NQ) in Australia experiences poorer health outcomes and a disproportionate burden of communicable diseases compared with urban populations in Australia. This study examined the governance of COVID-19 surveillance and response in NQ to identify strengths and opportunities for improvement.

**Methods:**

The manuscript presents an analysis of one case-unit within a broader case study project examining systems for surveillance and response for COVID-19 in NQ. Data were collected between October 2020–December 2021 comprising 47 interviews with clinical and public health staff, document review, and observation in organisational settings. Thematic analysis produced five key themes.

**Results:**

Study findings highlight key strengths of the COVID-19 response, including rapid implementation of response measures, and the relative autonomy of NQ’s Public Health Units to lead logistical decision-making. However, findings also highlight limitations and fragility of the public health system more generally, including unclear accountabilities, constraints on local community engagement, and workforce and other resourcing shortfalls. These were framed by state-wide regulatory and organisational incentives that prioritise clinical health care rather than disease prevention, health protection, and health promotion. Although NQ mobilised an effective COVID-19 response, findings suggest that NQ public health systems are marked by fragility, calling into question the region’s preparedness for future pandemic events and other public health crises.

**Conclusions:**

Study findings highlight an urgent need to improve governance, resourcing, and political priority of public health in NQ to address unmet needs and ongoing threats.

**Supplementary Information:**

The online version contains supplementary material available at 10.1186/s12913-023-10502-x.

## Background

The COVID-19 pandemic has underscored the critical importance of pandemic preparedness and health systems capability to support communicable disease surveillance and response in Australia and globally. Public health surveillance is the cornerstone of public health decision-making and practice and is fundamental to averting epidemics [1, 2]. Yet surveillance capacity, and pandemic preparedness, varies widely between and within countries [3]. Australia’s response to the COVID-19 pandemic led to comparative successes, by international standards, in transmission suppression, health system preparation, and control of case numbers; highlighting key strengths including effective coordination of response efforts across state and federal governments [4, 5]. In parallel, lessons from Australia’s pandemic response highlight opportunities to strengthen health system preparedness for future pandemics and other public health challenges [6].

**Fig. 1:**
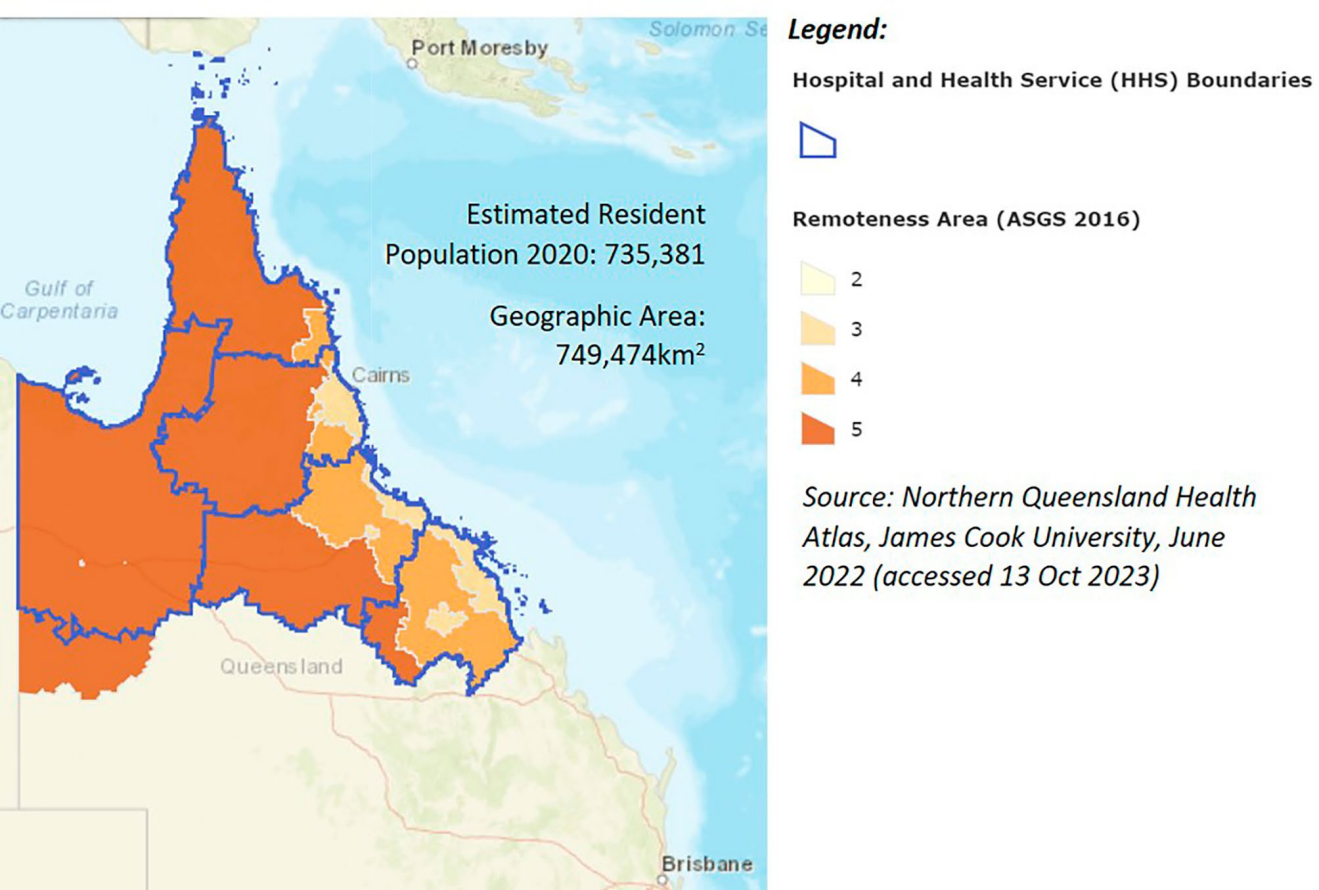
Hospital and Health Service (HHS) boundaries and Australian Statistical Geography Standard (ASGS) remoteness areas in northern Queensland

Public health surveillance in Australia operates at national, sub-national (state and territory) and local levels. Responses to health emergencies, including communicable disease outbreaks, are primarily the responsibility of state and territory health departments [7, 8]. Within the state of Queensland, northern Queensland (NQ) is a vast rural and remote region (Fig. 1) which lies within the Indo-Pacific geographic region. The region experiences poorer health outcomes and a disproportionate burden of communicable diseases compared with urban populations in Australia [9]. Unique geographic, demographic, and environmental conditions in NQ contribute to communicable disease emergence and outbreaks. There are many organisations, funding streams, programs, processes, people, and networks involved in COVID-19 surveillance and response in NQ. When the first COVID-19 cases in the NQ region were confirmed in March 2020, they occurred against a backdrop of concurrent communicable disease challenges and risks including incursion of mosquito-borne viruses (e.g., Japanese encephalitis) and exotic mosquito vectors, [10] an outbreak of syphilis, [11] and the threat of multi-drug resistant tuberculosis (TB) [12].

Theories of health system governance facilitate investigation of how different actors in a given system or organisation function and operate, with the aim of supporting health system strengthening [13]. Sheikh et al.’s health system framework recognises that any system – such as surveillance and response systems for COVID-19, is comprised of “hardware” and “software” components [14]. System hardware refers to tangible components such as financing, skilled health workforce, information systems, commodities and infrastructure that provide the material basis for service or surveillance [14]. System software refers to the values, norms, organisational cultures (the “usual way of doing business”), relationships and trust, and processes by which providers or managers are held to account – all of which transform material components into a functioning system [14]. To support pandemic response efforts and future pandemic preparedness, this study examines the governance of COVID-19 surveillance and response systems in NQ, with attention to both system hardware and software components, to identify key strengths and opportunities for improvement. In particular, the study examines: the role of formal rules (such as clinical protocols and guidelines, algorithms or operating procedures) and informal rules (such as workplace norms and organisational culture) in surveillance and response decision-making at different levels; and the relationships, trust and accountability mechanisms on which COVID-19 surveillance and response systems are enacted.

## Methods

This study is part of a broader project investigating communicable disease governance systems in NQ. The broader project adopts a single case study design with four embedded units of analysis; the current study reports on just one of those units, COVID-19 surveillance and response [15].

**Fig. 2:**
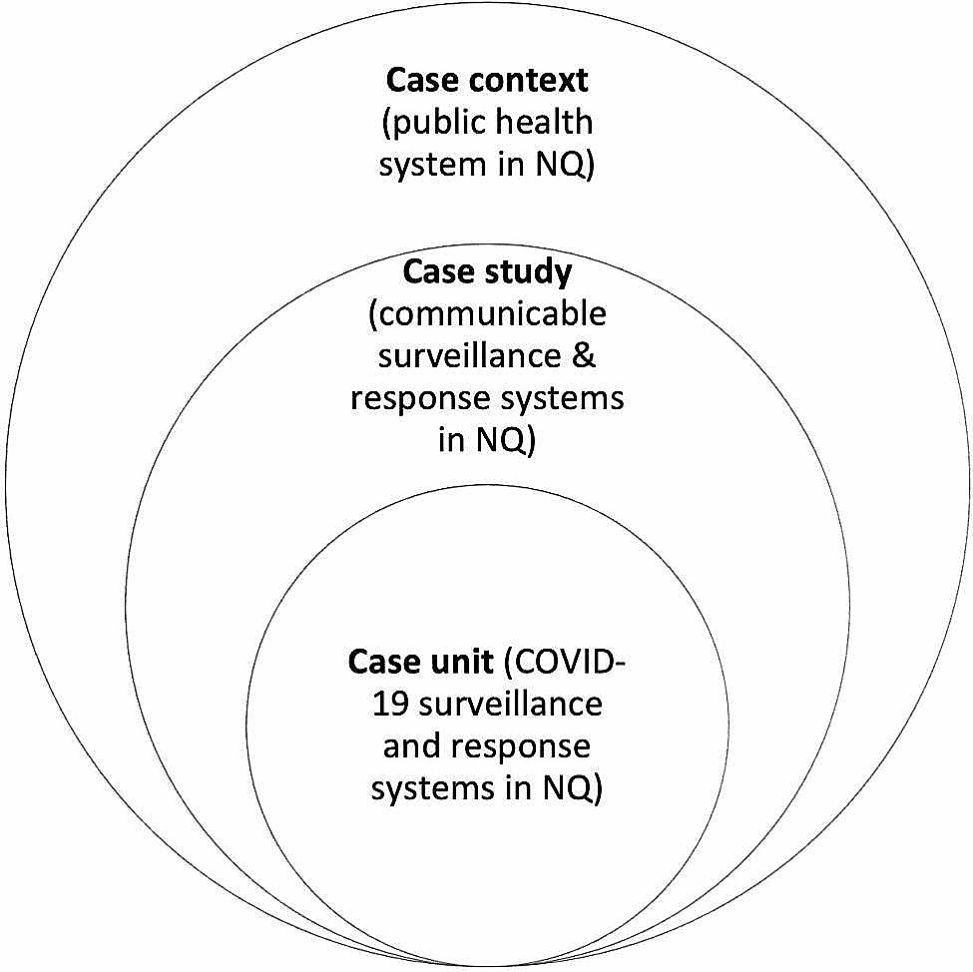
Case study and case context boundaries, showing the COVID-19 embedded unit of analysis

Case studies enable in-depth examination of complex issues in their real-world contexts. Embedded case study designs allow for one or more sub-units to be the focus of in-depth inquiry while allowing the broader topic to remain the phenomenon of interest [15]. The boundaries of the case are the communicable disease surveillance and response systems in NQ, with COVID-19 one of four embedded units of analysis in the broader study. The case study context is the broader public health system in NQ (Fig. 2). The four case units were selected for their differences according to organisational, biological, regulatory, and political factors; with the other case units being TB, mosquito-borne arboviruses and malaria, and sexually transmissible infections and blood-borne viruses.

**Fig. 3:**
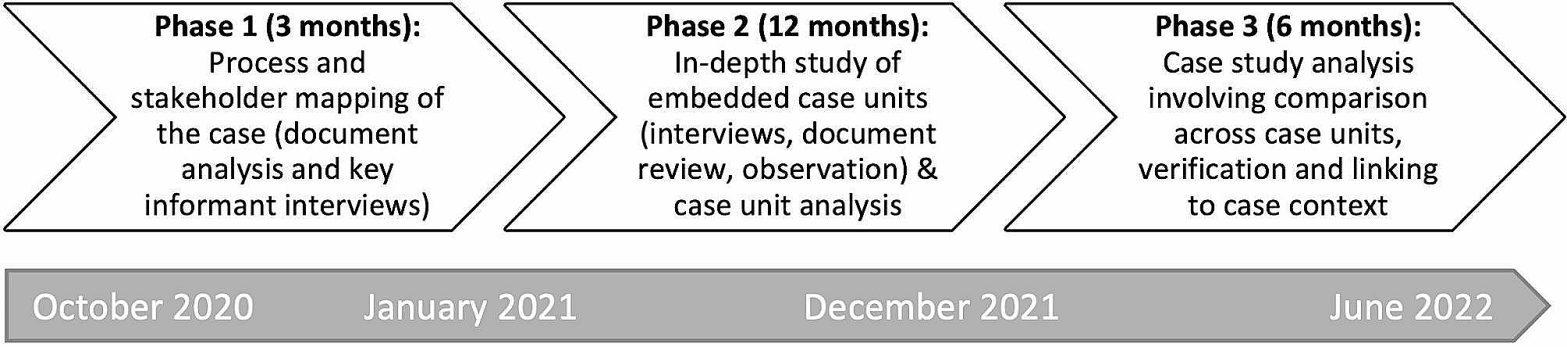
Three phases of the broader study

Data were collected and analysed between October 2020 and June 2022 across three project phases (Fig. 3). Phase 1 involved process and stakeholder mapping of the case predominantly via analysis of government and organisational documentation relating to communicable disease control in NQ. Three key informant interviews were also conducted with individuals occupying high-level roles within the NQ public health system. In Phase 2, a further 44 interviews were conducted to develop detailed case unit reports for each of the four embedded units of analysis. This involved selection of 10–20 individuals per unit of analysis, using both purposive and snowballing selection methods. Many aspects of the discussions with interviewees pertained to multiple diseases, case-level issues, and the case context.

Organisational charts from public websites and networks of the investigator team were used to identify potential interview participants occupying key roles at both front line and mid-level management within health care delivery and planning organisations in NQ. Both current and historical perspectives were considered. Role types (Table 1) of interviewees included: clinical staff such as infectious disease, emergency department physicians and pathologists; public health staff such as public health nurses, environmental health officers, public health medical officers, and epidemiologists; mid-level managers such as medical superintendents; and policy or research personnel holding policy, strategy and/or research roles in government or non-government organisations, including expert consultants. As the focus of the study is on the governance of communicable disease surveillance and response systems, which pertain predominantly to decision-making processes and platforms in key government and non-government organisations in NQ, interviews were not sought with patients or non-expert members of the community. An interview guide was developed for Phase 2 interviews (Appendix 1) with question fields relating to surveillance and response processes, workforce roles and relationships.

**Table 1:** Number of interviewees in Phases 1 and 2 of the study by category of professional role (n = 47)

Interviewee role type	Number of interviewees
Clinical (e.g., infectious disease and emergency physicians, pathologists)	7
Public health (operational & scientific roles – e.g., public health medical officers, epidemiologists, public health nurses, environmental health officers, entomologists, data analysts)	22
Mid-level managers or directors in health service organisations (e.g., Directors of Public Health Units, medical superintendents)	9
Policy/ research (e.g., staff/expert consultants in policy, strategy and/or research roles in government or non-government organisations)	9

Policy document review and (pre-approved) unstructured observations in the organisational setting of three public health offices within NQ Hospital and Health Services (HHSs) were also undertaken in this phase. Observations were conducted to identify key activities and relationships relevant to communicable disease surveillance and response within the main sites of public health decision making in NQ. This involved attendance over several days of one or two researchers (AE and ST) within busy office spaces and at routine meetings, and accompanying senior staff as they conducted their usual work. An observation template was used to prompt notetaking in the following fields: workplace description, roles, communication and relationships, nature of work, workflow characteristics, and tentative interpretations/theoretical memos. Documents were identified and collected from the websites of relevant organisations (e.g., HHSs, AICCHOs, government department websites) as identified by the research team and suggested by interviewees and included: published strategies and operational plans; annual reports; policy reports; federal-level and state legislation and regulations; health service contracts and agreements; performance frameworks; vision statements; action plans; and response/management handbooks.

Interviews from both the first and second phases were transcribed verbatim and were coded inductively in NVivo [QSR]. To develop the coding framework, two researchers (AE and SMT) coded a selection of four interview transcripts (one per unit of analysis) and met to compare approaches and decide on a suitable analysis framework. The resultant framework borrowed from the United States Centers for Disease Control and Prevention (CDC)’s 10 Essential Public Health Services [16], which reflect key public health processes enabling effective communicable disease surveillance and response, as high-level headings to group lower-level inductive codes that reflected both hardware and software elements. Document and observation data were used to create initial process and stakeholder maps and to support analysis of the interview data through triangulation. Thematic analysis produced case unit themes that reflect patterns of relationships between hardware and software components shaping surveillance and response processes and outcomes. Emerging findings from the work were discussed with individuals occupying roles at executive levels within health service organisations and in key policy roles within government entities. Notes from these meetings were used to verify and contextualise Phase 2 findings and link them to the broader policy context.

A subsequent Phase 3, of comparative analysis was conducted across all four embedded units of analysis, but is not reported here. This manuscript reports findings only from the COVID-19 case unit in Phase 2 of the study.

All methods were carried out in accordance with relevant guidelines and regulations including seeking informed consent from all interview participants. This project was approved by the Townsville HHS Human Research Ethics Committee (HREC) *HREC/2019/QTHS/59,811* with reciprocal approval received from the James Cook University HREC. Governance (site-specific) approval was received from the Townsville HHS, Cairns and Hinterland HHS, Torres and Cape HHS and Mackay HHS to enable data collection at these sites.

## Results

Findings are divided into two major sections. The first provides a descriptive account of shifts in regulatory and organisational settings in response to the pandemic in NQ, and their implications for public health activity in the NQ context. The second section reports thematic findings relating to the strengths of the approaches and the challenges experienced in their implementation, considering both hardware (material) and software (relational) factors.

### The NQ health system and features of the pandemic response

Public health services, or the preventive, protective and promotive functions of the NQ health system, are delivered by several organisations. Queensland’s 16 state-funded statutory entities called Hospital and Health Services (HHSs), five of which are located in NQ, are primarily funded to deliver secondary and tertiary health care services via hospital, clinic-based, and outreach services. Within the NQ HHSs, two Public Health Units (PHUs) in the Townsville HHS and the Cairns and Hinterland HHS deliver a range of services including disease notifications, screening services, vaccinations, vector control, and outbreak monitoring and response across the NQ region. The Townsville HHS PHU services its own region and the neighbouring North West and Mackay HHS regions, and at the time of study the Cairns and Hinterland HHS serviced its own region and the Torres and Cape HHS region. Health promotion services are also delivered by the PHUs, although this function was hampered by reductions to Commonwealth funding for health promotion, prevention and early intervention from June 2014 [17] and cuts made to the health promotion workforce resulting from a Queensland health service restructure and workforce reductions in 2012 and 2013 [18].

Primary care services in NQ are mainly delivered via private GP clinics, with the federally funded Primary Health Networks (PHNs) functioning as regional commissioning and planning bodies. A network of 12 Aboriginal and Torres Strait Islander Community Controlled Health Organisations (AICCHOs) are non-government organisations that deliver culturally safe primary care services supported by a peak body (the Queensland Aboriginal and Islander Health Council – QAIHC, which is a leadership and policy organisation that represents AICCHOs across Queensland).

On 29 January 2020, in response to the COVID-19 pandemic, Queensland’s Minister for Health and Minister for Ambulance Services declared *a public health emergency* under Sect. 319 of the *Public Health Act 2005 (Qld)*. The declaration initiated “command and control” [19] arrangements including activation of an emergency and disaster management policy infrastructure to frame the response, centred around Public Health Directions issued by Queensland’s Chief Health Officer (CHO) [20]. Like in other Australian jurisdictions, a wide suite of measures were introduced in Queensland to reduce COVID-19 transmission, including mask wearing in public, hotel quarantine, and home isolation orders. Biosecurity Zones, also known as COVID-19 Restricted Travel Areas or designated areas, were established in NQ under the *Biosecurity Act 2015* between March and June 2020. These Zones restricted entry to remote communities in Queensland for the purpose of slowing the spread of COVID-19 among community members at greater risk of becoming seriously ill if they contracted the virus [21]. In the Cape and Torres regions, the area north of Mossman and the Atherton Tablelands was a single zone, and in North West Queensland communities, Burketown, Palm Island, Doomadgee, and Mornington were also subject to high level restrictions. These restrictions heavily affected Aboriginal and Torres Strait Islander communities across NQ [22].

Rapid mobilisation of testing and surveillance systems in NQ included the use of point of care testing and reporting of cases through Queensland’s digital Notifiable Conditions Systems (NoCS). In the NQ HHSs, Health Emergency Operation Centres (HEOCs) were rapidly “stood up” to provide incident management support as part of the pandemic response. The AICCHOs and peak body (QAIHC) also developed and disseminated communication plans and public health messaging to communities about how to protect the community during the pandemic. In parallel, the roll-out of the COVID-19 vaccines commenced nation-wide in February 2021, largely led in Queensland by the HHSs. Logistical considerations for vaccine teams in the NQ HHSs included cold chain management, which involved planning, fridge audits and management of cold chain breaches.

As of September 2022, 233,139 confirmed cases of COVID-19 and 232 deaths were reported in NQ. These figures represented less than 1% of Queensland’s total cases and 11% of COVID-19 -related deaths [23]. The proportion of the Queensland population fully vaccinated was 93.1% in September 2022 [23].

### Key strengths and challenges

Key strengths and challenges are presented against five key themes: rapid implementation and coordination of local response measures; unclear lines of accountability; constraints on/gaps in local community engagement; workforce shortages and burnout; and lack of priority afforded to public health function in health services.

#### Rapid implementation and coordination of local response measures

A key strength of the COVID-19 response in NQ was the rapid implementation and coordination of logistical response measures locally from March 2020. Directed and framed by centralised powers associated with a state-wide declaration of a public health emergency in January 2020, the operationalisation of large components of the COVID-19 response fell to the two PHUs in NQ. As new Public Health Directions were released by Queensland’s Chief Health Officer, COVID-19 response teams in the PHUs rapidly translated broad advice into logistical activities involving local-level coordination with police, quarantine services, hotels, and a wide range of other stakeholders, in conjunction with testing and contact tracing activities led though the broader HHSs.

Region-wide responses were supported by collegiality and trust between senior public health staff in the HHSs. The two PHU directors and public health medical officers in the HHSs leveraged informal relationships and networks across the NQ region to share information, experiences and engage in localised response coordination, demonstrating the importance of health system software in enabling response efforts. Moreover, although a major structural reform and devolution process enacted in 2012 (involving the establishment of the HHSs) broke down many previous formal, region-wide communicable disease monitoring and response structures, the cultural legacy of the historical separation of public health services from local hospitals meant that the PHUs still operated with relative administrative autonomy from other local health structures in decision-making for aspects of the COVID-19 response. Despite PHUs being embedded in the HHSs for the purposes of broader (state-wide) management and resource allocation purposes (system hardware), this relative autonomy of PHUs at the local level allowed for rapid decision-making and operationalisation of the Public Health Directions, including proactive development of localised COVID-19 response plans.

*“So obviously, there’s a lot of state plans, but […] we’ve been very big on having localised plans as well […] we have pushed quite heavily for our HHSs and our PHUs to plan at least: ‘what would we do if we were in a situation where we needed more help [at a state level] and we couldn’t get it?’” Int 17*.

In parallel, and operating almost entirely autonomously from the HHSs, the AICCHOs and peak body representing AICCHOs in the state (QAIHC) rapidly developed and disseminated communications plans and public health messaging to communities about how to protect the community during the pandemic.

#### Unclear lines of accountability

Despite the rapid mobilisation of staff and resources locally, a recurring theme in participants’ accounts of COVID-19 surveillance and response systems was the lack of clarity in accountabilities framing the response. Although the quasi-independence of the PHUs within the HHSs offered a degree of autonomy as noted above, it also meant that Queensland’s Department of Health and HHS expectations of the role of the PHUs lacked clarity, alignment or was changeable.

*“We function in this little hybrid world where we’re part of the Hospital [and Health Service, i.e., HHS] but also separate from it. Because we were always separate from it and our structures and so forth have been maintained quite separately.” Int 17*.

In addition to reporting internally within their host HHS and delivering services to neighbouring HHSs, the PHUs also responded directly to separate divisions/branches within the Queensland Department of Health. During the COVID-19 pandemic an additional, and separate, disaster management reporting hierarchy was established. These overlapping reporting arrangements created an onerous administrative burden on the PHUs during the COVID-19 pandemic. For example, the local HHSs, and Queensland Department of Health’s Communicable Diseases Branch and COVID-19 Compliance Team, as well as the State Health Emergency Coordination Centre, all required regular collection and reporting by the PHUs of different, though related, COVID-19 data.

Despite these reporting requirements, the Queensland Department of Health provided little operational guidance to support planning and coordination of local pandemic responses in the PHUs. The Queensland Health Public Health Practice Manual (2016) legitimised this separation of operational and strategic responsibilities by casting the role of the Department as a high-level strategic and policy setting (rather than operational) body [24]. The Manual was introduced to provide clarity around accountabilities for public health following the devolution of responsibility for public health to the HHSs (from the Department of Health) from 2012; but in the COVID-19 response, this expectation of functional separation impeded two-way decision-making between the HHSs and the Department. Several participants expressed a struggle to balance a personal sense of responsibility for the success or otherwise of response measures locally, with an inability to participate in decision-making at a state level about what responses were needed and how they should be delivered. Top-down rules regarding communication and limited control of budgets (i.e. rules controlling system “hardware”) did not marry with the expectation and reality, that individuals demonstrate a strong sense of mission, adaptability and responsibility in the crisis (i.e. expectations around system “software”).

*“Technically if people [i.e., the Chief Health Officer and Department] are telling you what to do, they should supply you with the adequate advice on how to do it, [but] that’s probably the big gap […] It’s ‘you need to go do this’. Okay, well we’ve got all these issues. ‘Well, they’re operational, you [i.e., the PHU] just need to go work them out yourself. We don’t have the resources for that, that’s operational, you need to go and work those out yourself’.” Int 11*.

Consequently, local public health staff saw themselves as simultaneously accountable for response outcomes yet neglected in key governance processes and resourcing decisions that drove these outcomes, which contributed to feelings of vulnerability and frustration.

*“That’s one thing as well that I think I felt and all the [key staff in the PHUs] have felt with COVID - is the fact that they [the Queensland Department of Health] just bypassed us. And that also undermines your credibility and undermines your authority which doesn’t help really.” Int 14*.

Framed by the lack of clarity in accountability arrangements, several participants recounted poorly designed and executed responses in NQ. For example, one participant recounted that there were no processes established for review of biosecurity management plans for people accessing movement restriction exemptions; yet the mechanisms to raise and address this issue at a state level lacked responsiveness.

*“We had lots of people travelling in on [movement restriction] exemptions, which at that time, people on exemptions were meant to have biosecurity management plans most often than not, no one was reviewing and approving them […] and we saw gaps in it and we got onto SHECC [the State Health Emergency Coordination Centre] down in Brisbane and they’re like, ‘No one approves them. People just have to say that they’ve got one.’” Int 21*.

#### Constraints on/gaps in local community engagement

As part of the emergency response, new restrictions had been imposed by the Queensland Department of Health on HHS-led media liaison relating to COVID-19. Under the Queensland Public Health Sub-plan, [19] *Level 2 and Level 3 Events* require that all media releases and other public health awareness campaigns related to the event be endorsed by the State Health Coordinator (usually the Chief Health Officer or Deputy Director-General) prior to their release. Participants in the PHUs therefore described encountering restrictions on developing and sharing localised COVID-19 -related public health information with their communities.

“*The stuff we would normally do with an outbreak, we then weren’t able to do. We couldn’t do any localised comms. And we weren’t allowed to talk to the media.” Int 17*.

Participants also recounted a backdrop of staff cuts to community engagement roles in Queensland Health that followed the 2012 restructure, and low levels of trust between Queensland Health services and the AICCHO sector. The AICCHO sector represents a pivotal link with local communities; yet participant accounts and written reports indicated that there were few governance mechanisms in Queensland Health-led initiatives to involve the AICCHO sector in COVID-19 planning discussions, either at a local HHS level or with Department bodies. For example, it was reported that AICCHO services were not involved in the development of local pandemic plans, received little to no assistance or communication from HHSs throughout the initial crisis period, and were not consulted in relation to key policies including biosecurity zones or surge workforce [25]. Combined, these factors diminished the capacity of the PHUs to conduct local-level community engagement as part of the pandemic response.

Consequently, participants described that some COVID-19 -related policies and community education materials were not sufficiently responsive to local contexts and needs. For example, “fly-in-fly-out” models of vaccine implementation in some remote communities were described that had limited focus on improving Aboriginal and Torres Strait Islander community understanding of the vaccines (Int 15). Some participants also felt that local communities had not been effectively engaged in relation to the enactment of the Biosecurity Zones.

#### Workforce shortages and burnout

Workforce challenges identified in the study were framed by critical workforce gaps that pre-dated the COVID-19 pandemic. Participant accounts emphasised the significant public health workforce and workload implications of enacting COVID-19 surveillance and response activities at a local level, including to operationalise the Public Health Directions in the PHUs. Yet, the availability and deployment readiness of a “surge” workforce in the HHSs was limited.

*“The whole thing about how you plan to ramp up contact tracing in the event of a big state-wide or local event is still a struggle because we’ve got a list of people who have been – who are existing, or could be trained as, contact tracing officers. But then getting them released, particularly in circumstances where the hospital is not allowed to turn around and reduce services, is almost impossible. Unless they [HHS administrators] know that it’s a critical staff member, you can’t have them.” Int 15*.

The difficulties mobilising a surge workforce meant that the increased workload burden required to support the COVID-19 response largely fell to existing public health services and teams within the Cairns and Townsville PHUs. In at least the first 12 months of the pandemic, a large proportion of staff in the PHUs were re-allocated – in either full or part-time capacities, or simply on top of other work – to the COVID-19 response from vector control, sexual health, communicable disease control and other business as usual (“BAU”) public health services. In the Townsville PHU at the time of data collection, for example, the vector control team had been entirely re-directed towards assisting with hotel quarantine. Similar disruptions were described in other vector control and BAU activities across NQ.

*“We just have to figure out what needs to be sacrificed. And unfortunately, that would probably have to be a lot of BAU [business as usual] stuff, and so only follow up the most urgent cases which is pretty sad.” Int 18*.

Emergency COVID-19 funding enabled the establishment of some new positions in the HHSs to conduct testing, contact tracing and follow up. However, this funding was time-limited, meaning that employment arrangements for new staff were short-term. On top of delivering critical COVID-19 response functions, staff in the PHUs described having to continually re-apply to HHS administrators for funding every three months to support the new positions needed for the ongoing COVID-19 response, causing planning problems and compounding recruitment challenges. As the emergency funding ceased, participants described an erosion of redundancy capacity, meaning an inability among staff remaining involved in the response to step away from their roles even for short periods. As people returned to previous roles, the remaining teams were smaller yet there had not been a concomitant decrease in workload or reduction in “high alert intensity” (Int 17).

The intense workload pressures experienced by public health staff had led to increasing feelings of fatigue and burnout during 2021, eroding motivation to maintain or build relationships (system software) that were essential to many COVID-19 specific and more routine public health functions. Moreover, these experiences were framed by a sense among participants that public health continues to be generally poorly understood, and little valued, by health service administrators whose decisions are central to perceptions of under-resourcing (system hardware) of public health personnel and activities.

*“At an administrative level, the hospital […] doesn’t make any allowance for public health being different to how the rest of the hospital works […] one of the big objectives is to provide patient centred care. Of course, public health is not about patient centred care, but everything has to be about patient centred care because that’s the flavour of the current [HHS] strategic plan.” Int 15*.

#### Lack of priority afforded to public health function in health services

The final theme reflects a widespread concern among participants that public health generally lacks priority – in terms of both values (software) and resourcing commitments (hardware) – within the HHSs. Participants recounted a need, both pre and during COVID-19, to explain and defend the value of public health as a service directorate to HHS administrators. While some felt that the COVID-19 response may have drawn attention to the value of public health (“*Public health […] is sexy all of a sudden” Int 43)* others pointed to a hospital-centric approach in the way that COVID-19 emergency funding was overwhelmingly used to support frontline clinical, rather than public health, activities in the HHSs during the study period. One participant recounted that, despite state and federal COVID-19 resourcing including substantial increased support for contact tracing and testing activities, COVID-19 response plans tended to be reactive: demonstrating an underlying, persisting lack of understanding of critical prevention activities.

*“One of the difficulties we’ve had is people understanding that public health needs to respond before the curve, not with the curve. Hospitals respond to demand. Public health needs to respond to risk.” Int 17*.

Structurally, there are no key performance indicators in the HHS Service Level Agreements that map to public health (preventive, protective, and promotive) priorities, and a very small proportion of HHS budgets is allocated to public health services. Allocations for *Prevention Services – Public Health* in the Service Level Agreements *(2019/20-2021-22)*, for example, represent only between 0.1 and 2% of HHS non-capital allocations [26]. Moreover, participants described a lack of transparency in these budget allocations within the HHSs, and the challenge of relying on temporary project-based funding to support several critical public health functions.

*“So what is killing us more than anything else is understaffing and temporary funding from multiple pots that we don’t understand. I don’t even understand the slice of the HHS budget that is available to us. We only pretend to budget here.” Int 15*.

## Discussion

Australia has a robust national regulatory architecture for communicable disease surveillance and response; yet identifying gaps and opportunities to strengthen overall preparedness for infectious disease outbreaks and pandemics requires detailed investigation of localised capacities, activities and relationships. This COVID-19 case unit, nested within a broader case study on communicable disease surveillance and response systems in NQ, highlights several critical insights to inform health system strengthening in a region with higher communicable disease risks and unmet health needs. Study findings highlight key strengths of the COVID-19 response, including rapid implementation of response measures, and the relative autonomy of the PHUs to lead logistical decision-making. However, the study also reveals critical limitations of the public health system in NQ, including unclear accountabilities, constraints on local community engagement, and workforce and other resourcing shortfalls – framed by regulatory and organisational elements prioritising clinical health care rather than disease prevention, health protection, and health promotion. We reflect on what these experiences demonstrate with respect to Queensland’s public health surveillance and response capacity, not only in the pandemic but more broadly.

First, the findings highlight an urgent need for prioritisation of public health within Queensland’s decentralised health system. Public health (as distinct from publicly funded health services) is concerned with the protection and promotion of health, and prevention of injury, illness, and disability. It is a core responsibility of government and conceptualised as an integral component of comprehensive primary healthcare; yet our study findings indicate that this distinction is poorly understood in the devolved organisational context of the HHSs in NQ and potentially beyond, even accounting for the experience of COVID-19. Others have criticised the clinical / biomedical orientation of Australia’s health system and the urgent need for policy redirection towards public health, [27] as well as the need to clarify definitions, terminologies and classifications to identify what is a “public health” activity and facilitate comparison of this activity across jurisdictions [28]. In Queensland, the organisational position of PHUs – nested within and dependent for resources on board-governed health districts with typically hospital-focused administrators – is poorly suited to public health decision-making. Gaps in support for public health capacity in NQ was a key finding of a Queensland Government review of COVID-19 response, [6] underpinning a recommendation to amend legislation to strengthen accountability for population health in Queensland Health. Delivering on this recommendation needs to be accompanied by adequate resourcing for key workforce roles to support a full complement of public health functions across the diverse NQ region. Without this, pandemic responses will remain overwhelmingly reactive, rather than responsive to risk.

Second, the study highlights opportunities to proactively explore public health workforce models that are amenable to scaling up and down to support an effective response, supported by foundational, effective, and permanent public health services in the HHSs. The study demonstrates that emergency funding from a low-capacity base is not sufficient to deliver a sustained public health response: our study identified only modest increases in budgets relative to new responsibilities, inclusive of developing and managing a hotel quarantine system from scratch. The PHUs across the NQ region faced workforce shortages and burnout resulting in significant disruptions to other longstanding public health priorities including sexual health and vector management for arboviruses. The full implications of these workforce redirections globally are yet to be seen, but there are fears that disruptions to prevention and treatment services during the first two years of the COVID-19 pandemic will drive higher case numbers of other diseases such as TB [29].

Third, the study highlights critical gaps in community engagement capacity within NQ public health systems. While this to some extent reflects the emergency response (“command and control”) approach as well as capacity and resourcing constraints in the PHUs, it may also reflect gaps in capabilities and workforce models within the HHSs to proactively lead engagement with diverse communities across the NQ region. The gaps evident in the current study in the relationship between Queensland Health and the AICCHO sector point to an urgent need for relationship and trust-building to support coordination of community engagement activities and surveillance and response services for NQ Aboriginal and Torres Strait Islander populations. The Queensland Health/QAIHC 2021 joint *Health Equity Framework* [30] outlines a strategic framework to drive health equity, and eliminate institutional racism across the health system as a foundation for improving health outcomes for Queensland Aboriginal and Torres Strait Islander peoples. Future planning for community engagement in public health in NQ must be guided by this Framework. There may also be opportunities to strengthen resourcing of local community engagement and feedback mechanisms through emphasising these activities in future pandemic plans.

Overall, although NQ is regarded to have mobilised an effective COVID-19 response, [6] the findings of the study highlight that NQ public health systems are marked by fragility, calling into question the region’s preparedness for future pandemic events and other public health crises. The findings of this component of the study highlight that to strengthen public health systems in NQ there is an urgent need to improve clarity in accountability relationships for PHUs, strengthen coordination between services, invest in the public health workforce, and improve the responsiveness of services to local need through mechanisms supporting effective community engagement. There is also an opportunity for ongoing research to strengthen the case for public health investment in NQ, state-wide and nationally, by highlighting the overwhelming health benefits and cost savings attributable to responding to risk rather than demand.

Key strengths of the study include its attention to both “hardware” and “software” health system elements to identify key strengths and challenges, and the use of multiple data sources to enable data triangulation. Limitations include the focus of the study on state government-funded services as the entities with legislated responsibility for public health in Queensland. In addition, there were some limitations to the US CDC’s 10 Essential Public Health Services as an analytic framework including limited guidance or attention to critical domains in a public health crisis, such as post-disaster recovery, surge capacity (beyond workforce), and continuity of essential services. As fewer interviewees were included from the primary care sector, future governance-focussed research might also seek to explore structures and networks established to support communicable disease surveillance and response functions outside of state-funded services. Patients and the broader public were not represented in the current study whose focus was on governance systems and their key institutions. As critical stakeholders, however, future work should be conducted to consider the perspectives and experiences of patients and the broader public regarding issues of public health governance.

## Conclusion

Study findings highlight key strengths of the COVID-19 response, including rapid implementation of response measures, and the relative autonomy of the PHUs to lead logistical decision-making. However, the study also reveals critical limitations of the public health system in NQ, pertinent to longer term aspirations for pandemic preparedness in Australia. First, the findings highlight an urgent need for prioritisation (both strategic and operational) of public health within Queensland’s decentralised health system. Second, the study demonstrates opportunities to proactively explore public health workforce models that are amenable to surge mobilisation for effective outbreak and pandemic responses. Third, the study highlights critical gaps in community engagement capacity within NQ public health systems, underscoring the importance of approaching future community engagement planning from a foundation of trust between key NQ services. Overall, the findings of the study demonstrate an urgent need for improved governance, resourcing, and political priority of public health in NQ to address unmet needs and ongoing pandemic, and other public health threats.

### Electronic supplementary material

Below is the link to the electronic supplementary material.

Appendix 1: Phase 2 - interview guide

## Data Availability

Due to confidentiality agreements, qualitative de-identified data from interviews conducted during the current study are only available from the corresponding author on reasonable request to bona fide researchers. Other data derived from public resources are made available in the article.

## Abbreviations

AICCHO: Aboriginal and Torres Strait Islander Community Controlled Health Organisation(s)
HEOC: Health Emergency Operation Centre(s)
HHS: Hospital and Health Service
PHU: Public Health Unit
NoCS: Notifiable Conditions Systems
NQ: North Queensland
QAIHC: Queensland Aboriginal and Islander Health Council
