# Supplementary material for: “Hospitals respond to demand. Public health needs to respond to risk”: health system lessons from a case study of northern Queensland’s COVID-19 surveillance and response
Source: BMC Health Serv Res. 2024 Jan 18;24:104. doi: 10.1186/s12913-023-10502-x (PMC10797896; doi:10.1186/s12913-023-10502-x)
Supplement: Supplementary file 1 — Appendix 1: Phase 2 - interview guide [file 12913_2023_10502_MOESM1_ESM.docx]

**Appendix 1: Phase 2 – interview guide**

***Background***

Many thanks for agreeing to participate in this interview.

1. Could you please introduce yourself and your current role?
2. Can you describe what involvement and responsibilities you have for surveillance and/or response to [disease area] in your current role?
3. How important do you believe surveillance and response to [disease area] are for Queensland specifically and Australia more generally? [why]

***Service delivery***

1. From your perspective what are they key features and strengths of the current [disease area] surveillance systems in north Queensland?
2. What are the key pathways and mechanisms currently in place to respond to cases or outbreaks of [disease area]?

***Health workforce***

1. Which organisations, and which individuals would you say play critical roles in the current system? [consider all phases: data collection; data integration; analysis and interpretation; public health action; dissemination; system evaluation]
2. Thinking about key [disease area] response pathways, are there any individuals whose role is particularly important to the maintenance and function of effective response planning or implementation?
3. Thinking broadly, what would you say are the human strengths and weaknesses of our response system for [disease area]?

***Financing, health information systems, and leadership and governance***

1. In your experience, how do issues of trust and inter-organisational / jurisdictional relationships influence surveillance and response for [disease area]? [explain]
2. Based on your experience, are there any areas of the surveillance and response systems in north Queensland and Australia more broadly you would like to strengthen? [eg: resourcing; interoperability; governance; information systems]? What would you see as a priority?

***Other thoughts/gaps***

1. Is there anything else that you would like to add in relation to your experiences in surveillance and response for [disease area]?
